# Ocular kinetics and safety of intravitreally injected angiotensin converting enzyme inhibitor lisinopril

**DOI:** 10.1186/s40942-018-0146-7

**Published:** 2018-11-14

**Authors:** Madhu Nath, Nabanita Halder, Parijat Chandra, Sundararajan Baskar Singh, Ashok Kumar Deorari, Atul Kumar, Thirumurthy Velpandian

**Affiliations:** 1 Department of Ophthalmology, Dr. Rajendra Prasad Centre for Ophthalmic Sciences, All India Institute of Medical Sciences, New Delhi, 110029 India; 2 Ocular Pharmacology and Pharmacy Division, Dr. Rajendra Prasad Centre for Ophthalmic Sciences, All India Institute of Medical Sciences, New Delhi, 110029 India; 3 Department of Biophysics, All India Institute of Medical Sciences, New Delhi, 110029 India; 4 Department of Neonatal Intensive Care Unit, Pediatrics, All India Institute of Medical Sciences, New Delhi, 110029 India

**Keywords:** Lisinopril, Intravitreal injection, Ocular safety, Electroretinography, Pharmacokinetics

## Abstract

**Background and objectives:**

The study investigated the intravitreal safety and vitreous disposition of lisinopril, an angiotensin converting enzyme inhibitor in rabbits for its projected use in retinopathy.

**Methods:**

For the safety study, following the baseline ERG recording and fundus photography, 40 µg/50 µl of lisinopril sterile injection was injected unilaterally in the rabbit eyes (n = 4), where other eye served as a control. The electroretinogram and fundus images were obtained at 24, 48, 72 and 168 h following the intravitreal injection. For pharmacokinetics evaluation of the lisinopril, one eye of each rabbit (n = 4) received an intravitreal injection of lisinopril (40 µg/50 µl). The concentration of lisinopril in the ocular tissues, humours, plasma, lung, kidney and liver were measured through ESI-LC-MS/MS.

**Results:**

Upon the electroretinography studies, no significant difference was observed in the ERG pattern in the lisinopril injected eye when compared to the baseline of the respective animals till the 7th day of the study. In the fundus imaging, no morphological changes were observed in the retina of the animal. The concentration of the lisinopril was found to be above to the IC50 in the retina-choroid till 36 h. The concentration found in the plasma and body tissues were many folds less than the IC50 of the lisinopril.

**Conclusions:**

Intravitreal injection of 40 µg/50 µl of lisinopril found to be safe in the rabbit eye as evidenced by the electroretinography and fundus imaging studies. The average half-life of lisinopril is 12.6 h and the above-mentioned dose able to sustain its IC50 value till the 36 h.

## Background

Renin angiotensin system is reported to have local synthesis in the eyes, lungs and liver [1]. The possible role of the renin angiotensin system in these tissues is to maintain the local vascular tone and tissue homeostasis. Over-expression of this system has known to induce the pathology in the organs it is been secreted locally [1]. Ocular renin angiotensin system has been explored for their involvement in the various ocular pathologies such as diabetic retinopathy, glaucoma, ocular hypertension, neurodegeneration and retinopathy of prematurity [2–5].

The role of retinal renin angiotensin system in the ischemic retinopathy became more apparent after some major clinical trials end point data results. The EUCLID Study enrolled the diabetic patients observed that the angiotensin converting enzyme (ACE) inhibitor lisinopril reduced the risk of progression of retinopathy by approximately 50%, and also significantly reduced the risk of progression to proliferative retinopathy [6]. These findings were consistent with evidence that the renin-angiotensin system is expressed in the eye, and that adverse effects of angiotensin II on retinal angiogenesis and function can be inhibited by ACE inhibitors or angiotensin II-receptor blockers [7, 8]. In 2005, Sarlos and Berka has shown that in the rat developing retina RAS components were found in retinal blood vessels and cells of the ganglion cell layer, where it may influence the early stages of vascularization [9]. It has also been experimentally shown that the blockade of the renin-angiotensin system, not only targets pathological angiogenesis but also promotes re-vascularization of the retina and are likely to prove important in the treatment of those suffering from retinopathy of prematurity [10].

The clinical and experimental studies have revealed that ACE inhibition could be a potential target for arresting the progression of the retinopathy in diabetes and retinopathy of prematurity [8, 10, 11]. But, the systemic administration of ACEI might bring adverse effect in neonates suffering from ROP, also intravitreal administration will be a more targeted approach in resolving ischemic retinopathy.

As there is a lacunae about the safety of intravitreal administration of ACEI in ischemic retinopathy, the present study was carried out in order to evaluate the toxicity, safety and pharmacokinetics of angiotensin converting enzyme inhibitor lisinopril. The study would be beneficial in providing the safety profile of the drug in the ischemic retinopathies such as diabetic retinopathy and retinopathy of prematurity.

## Methods and materials

This study was conducted in accordance with ARVO guidelines; the protocol (689/IAEC/12) was approved by the institute animal ethics committee (All India Institute of Medical Sciences, New Delhi, India). Lisinopril was obtained from Sigma (USA). For electrophysiology and fundus imaging studies, rodent ocular imaging system fitted with rabbit eye objective lens (MIRON III, Phoenix Research Laboratories, USA). Other chemicals used were of analytical grade obtained from the respective commercial vendors.

### Preparation sterile intravitreal drug formulation

Lisinopril sterile injection was formulated for intravitreal use at the High Sterile Pharmaceutical Facility of the Ocular and Pharmacology Pharmacy Division of Dr. RPCentre for Ophthalmic Sciences by a qualified Pharmacist. Briefly, accurately weighed lisinopril (pharmaceutical grade) was dissolved the measured amount of sterile saline in the clean room, the resulting solution was mixed well and passed through 0.22 µm sterile filters (Milex, Millipore, USA) to remove any particulate matter. After checking it for pH, it was filled, sealed in glass ampoules and subjected for autoclaving. Drug concentration (0.08% w/v) in the prepared ampoules was confirmed by LC-MS/MS (liquid chromatography tandem mass spectrometry), and sterility was confirmed by microbiology before approving them for injection.

### Intravitreal kinetics of lisinopril

New Zealand rabbits, weighing (1.5–2 kg) were used in the study. Rabbit’s ocular surface was anesthetised using the proparacaine hydrochloride 5% ophthalmic solution. Prior to intravitreal injection, eyes were washed with 5% povidone-iodine, and 50 µL (40 µg) of the sterile lisinopril solution (0.08% w/v) was injected into the mid-vitreous in the left eye through pars plana route. The rabbits (n = 4 each time point) were euthanized with carbon-di-oxide at 1, 2, 8, 24, 36 and 48 h. Eyes were enucleated and stored at − 80 °C. The vitreous was dissected free from the frozen eyes using the technique described by Able and Boyle (1976), the aqueous humor was also separated during the dissection of the frozen globe [12]. The cornea was excised in the limbus and the retina with choroid was carefully dissected from the sclera using the method adopted by Gupta et al. (2000) [13]. Ocular tissues were collected and stored at − 80 °C till the analysis of lisinopril concentration through LC-MS/MS method described below.

### Plasma and body tissue levels after intravitreal injection of Lisinopril

Following intravitreal injection 40 µg/50 µL of lisinopril into the mid-vitreous in the left eye vitreous cavity through pars plana route, the rabbits (n = 4 each time point) were euthanized with carbon-di-oxide at 1, 2, 8, 24, 36 and 48 h. Lung, kidney and liver were collected and stored at − 80 °C till the analysis through LC-MS/MS method described earlier. Blood was also collected by cardiac puncture in EDTA vials and plasma separated by centrifugation and stored at − 80 °C until analysis.

### Sample preparation for tissues, humor and plasma

All weighted tissues were cut into small pieces and were homogenized using 95% methanol with 0.1% formic acid (spiked with 50 ng/ml internal standard) using polytron homogenizer. The tissues, plasma and humor extracts were subjected for centrifugation at 8500 g for 10 min. The supernatant was collected and 5 µl was subjected injection through auto sampler for quantitative analysis using the LC-MS/MS method mentioned below.

### ESI-LC-MS/MS method for the quantification of lisinopril

Lisinopril presence in plasma, ocular humours and tissues were estimated using positive liquid chromatography (Surveyor, Thermo, USA) coupled Electrospray ionization tandem mass spectroscopy coupled tandem mass spectroscopy (4000QTrap, Absciex, USA).

Briefly, the analytical separation of both the compounds was achieved using a phenyl-hexyl column (Merck, Germany) using a gradient elution with water (with 0.1% formic acid) and methanol (Merck, Germany) at the flow rate of 200 µl/min to elute both analytes simultaneously. Sulfadimethoxine (SDM) was used as the internal standard. For the quantification, multiple reaction monitoring mode was used with the transitions of 406.1 246.1, 309.5 (transition method) and 311/129 for lisinopril and sulfadimethoxine respectively. All other source and compound dependent parameters were optimized using the inbuilt algorithm to get maximum ions intensity in the analysis to reach the required sensitivity. Appropriate spiked standards were used for the construction of a calibration curve for the calculation of unknown concentration using the inbuilt algorithm of Analyst Ver. 1.5.2.

### Ocular safety study after intravitreal administration

New Zealand rabbits (n = 4), either sex, weighing (1.5–2 kg) were used in the study. Baseline electroretinogram (ERG) was obtained for rabbits prior the intravitreal injection as the method described below. Rabbit’s ocular surface was anesthetised using the proparacaine hydrochloride 5% ophthalmic solution. The eye was washed with povidone iodine, and 40 µg/50 µL of lisinopril was injected in the left eye through intravitreal injection at 1.2 mm away from the limbus, after anterior chamber tapping under aseptic condition. The effective dose was calculated from the previous study performed in our lab [8]. The retina was evaluated for the possible toxicity post intravitreal injection of lisinopril at 24, 48, 72 and 168 h using ERG study.

### Scotopic electroretinogram (ERG) protocol

The electroretinogram study protocol for evaluating the retinal toxicity was standardised in our lab. The rabbits were anesthetised using 25 mg/kg ketamine and 5 mg/kg xylazine. Eyes of the rabbit were dilated using tropicamide 0.8% and phenylephrine 5%. The corneal electrode (gold) was in touch with the centre of the cornea. The ground electrode was connected to the tail of the animal. The reference electrode was placed on the forehead. The ERG responses were obtained through ERG attachment of MICRON III rodent imaging system using Labscribe software (Phoenix laboratory, USA) and were performed according to ISCEV guidelines. The retina was stimulated using the white light of 1 cds/m^2^ intensity; the mean of 25 averages was taken for obtaining single ERG response from rabbit retina.

### Fundus imaging

Following Electroretinogram (ERG) study, rabbit retina was focused and fundus images of both eyes were recorded using streampix software using MICRON III rodent imaging system (Phoenix Lab, USA) after subjecting them for ERG.

### Calculation and statistical analysis

The ERG waves were analysed by the amplitude and latencies of ‘a’ and ‘b’ wave. The ‘a’ wave amplitude was calculated as the amplitude at the beginning point of ERG to the hyperpolarised trough, whereas the ‘b’ wave amplitude was calculated as the maximum point of the crest from the ‘a’ wave amplitude. The latencies of ‘a’ and ‘b’ were calculated as the time from the initiation of ERG wave to the negative notch of ERG wave time (‘a’ wave latency) and initiation of ERG to the maximum point of the hyperpolarised wave (‘b’ wave latency). The amplitude and latency were expressed in microvolt and millisecond respectively.

A commercially available statistical software package (SigmaPlot ver. 11 for Windows, Systat Software Inc. USA) was used to perform statistical analysis of the data. Data are presented as mean ± SEM. Intergroup analysis was performed using unpaired Student *t* test and non-parametric Mann–Whitney rank sum test when appropriate and a *p* < 0.05 was considered as significant.

## Results

### Preparation of intravitreal lisinopril formulation

**Fig. 1:**
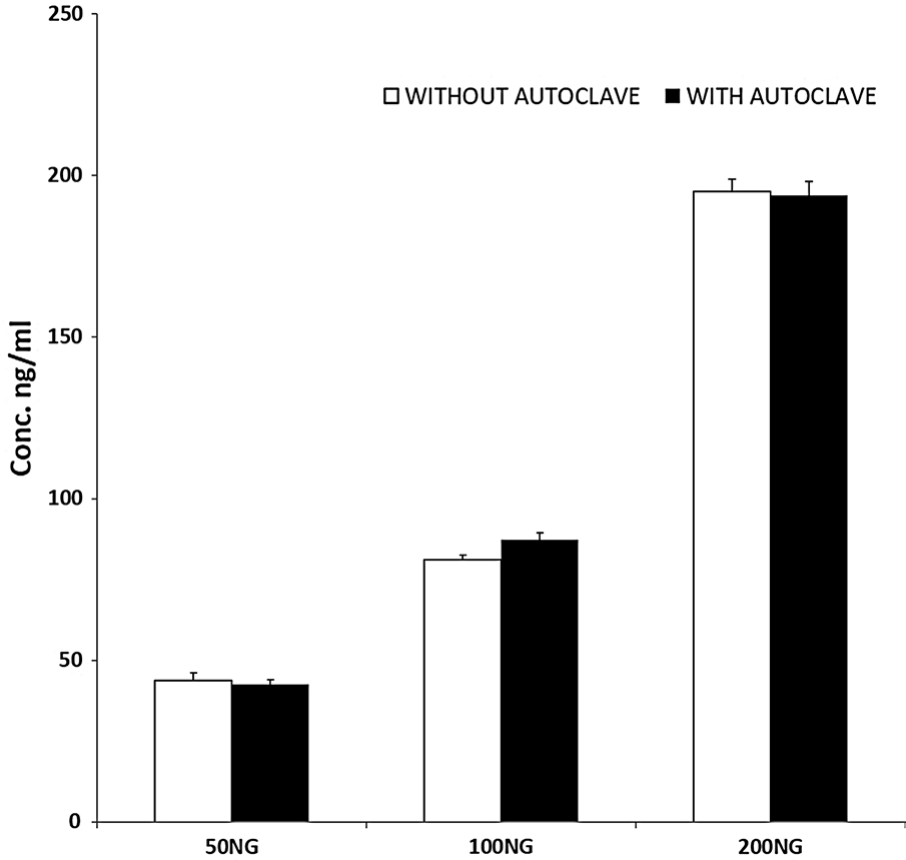
The concentration of lisinopril, before and after subjecting the formulation for sterilization. No significant difference was found

The intravitreal formulation of lisinopril was found to be stable after thermal sterilization by autoclaving. The data shown the concentration of lisinopril before and after subjecting the formulation for sterilization is shown in the Fig. 1.

### Intravitreal kinetics of lisinopril

**Fig. 2:**
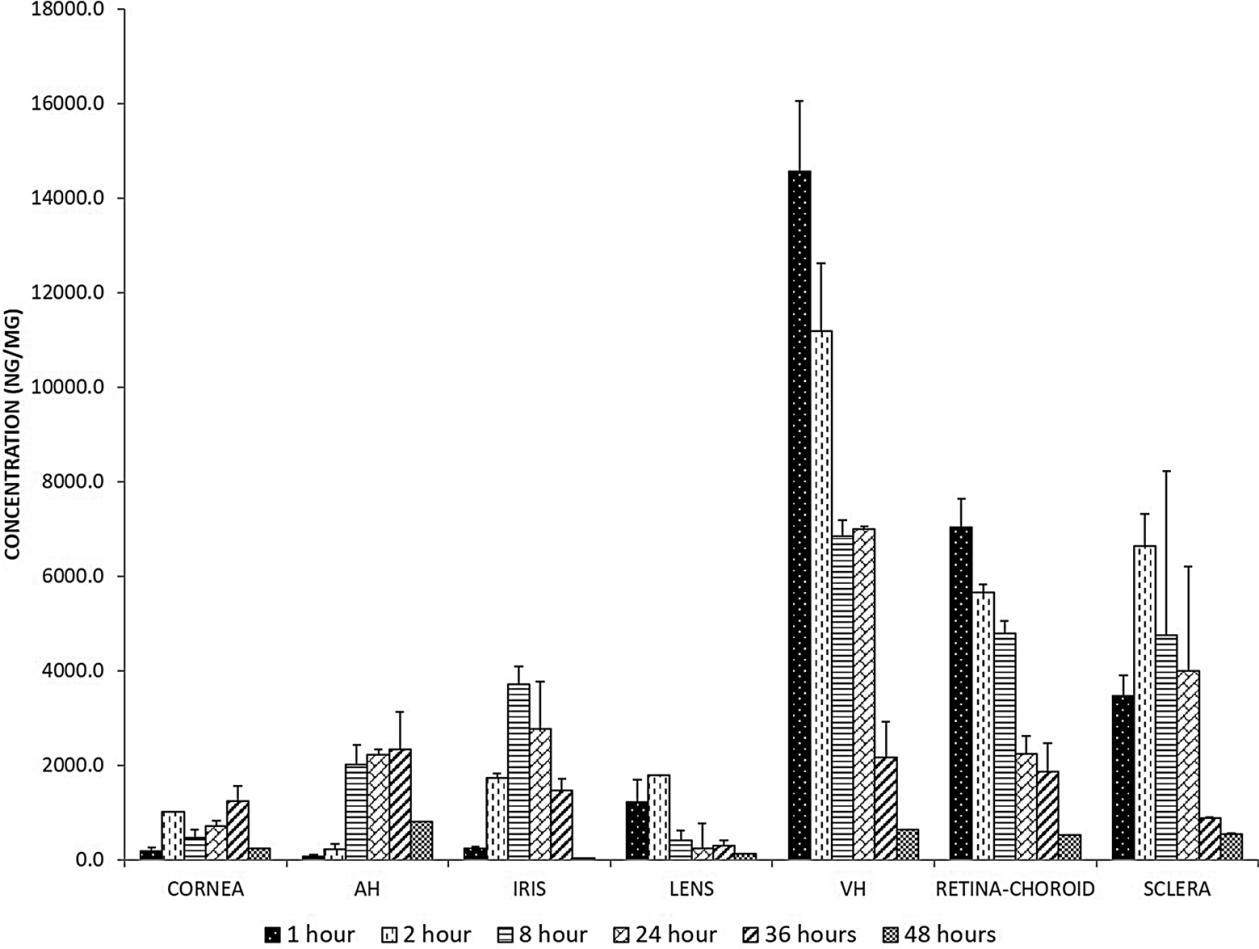
The lisinopril levels in the ocular humours and tissues. The concentration is represented as ng/ml. Data are represented as mean ± SEM

The sterile formulation was unilaterally injected in the rabbit eye. The lisinopril concentration in the vitreous humour (14.52 µg) and retina-choroid (7 µg) found to be highest at 1 h. The drug concentration remained above IC50 till 36 h in the vitreous and retina choroid. The concentration in sclera reached its maximum concentration (6.6 µg) at 2 h. The drug concentration increased steadily in the anterior part of the eye at later time points (Fig. 2).

### Plasma and visceral tissue concentration

**Fig. 3:**
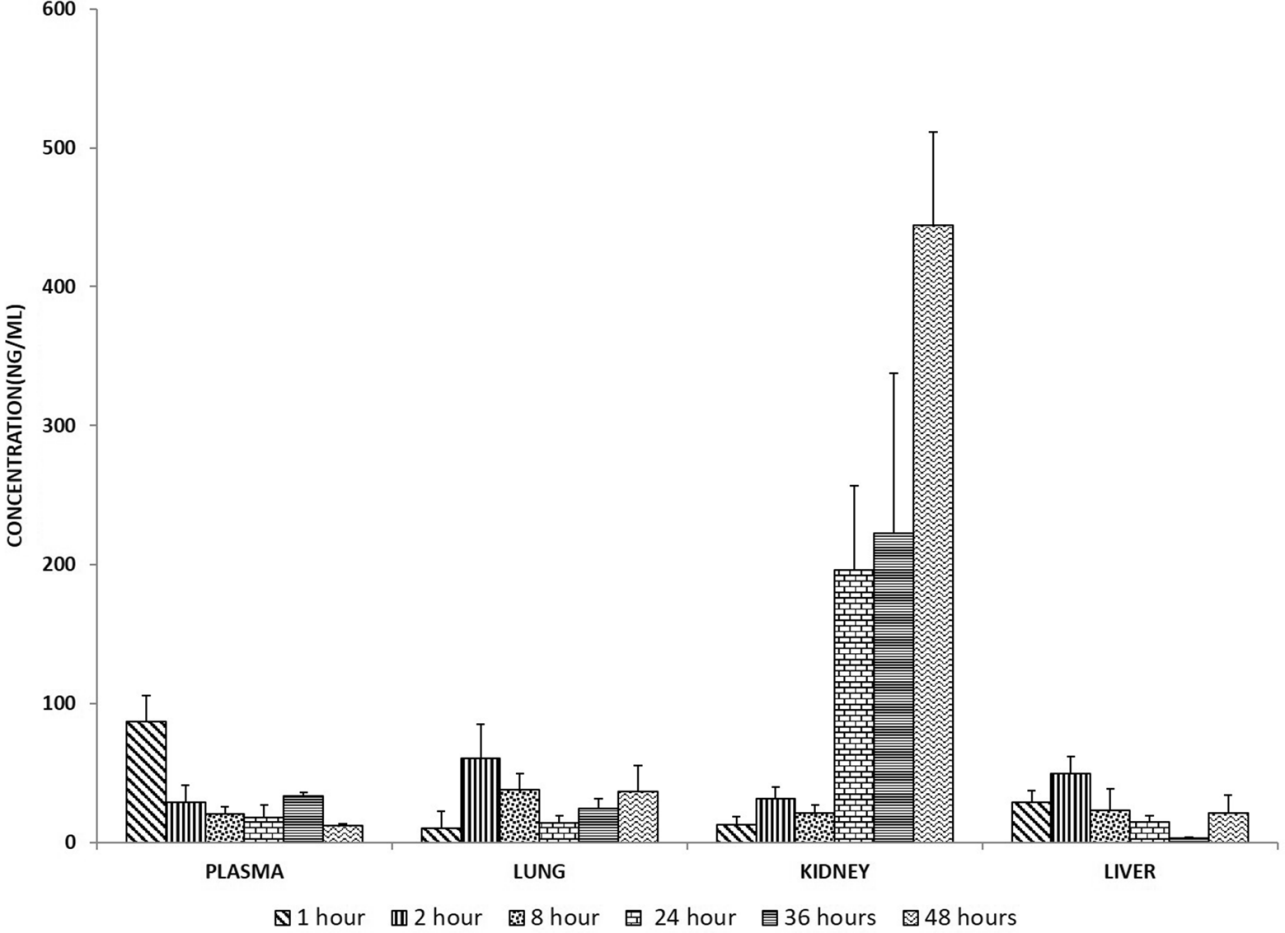
The lisinopril levels in the plasma, lungs, kidney and liver. The concentration is represented as ng/ml. Data are represented as mean ± SEM

Following intravitreal injection, lisinopril was able to leach out in the systemic circulation, but a very low concentration of lisinopril was detected in the plasma (87.1 ng/ml) at 1 h which declined to 12 ng/ml up to 48 h (Fig. 3). The concentration of lisinopril was found to be increased in the kidney by the end of the 48 h. The concentration in liver and lung were also found to be less than the IC_50_ of lisinopril.

### Functional assessment of retina

**Fig. 4:**
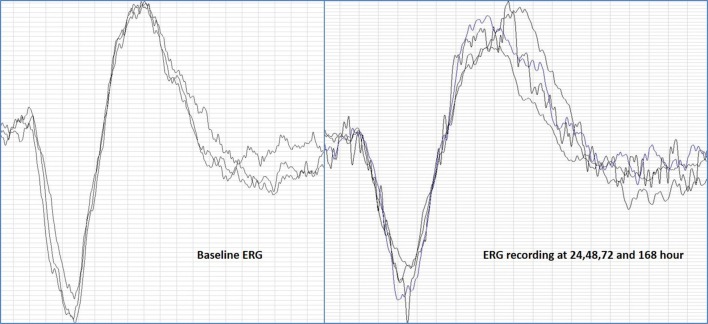
Fig represents the baseline ERG and the ERG waveform of 24, 48, 72 and 168 h. The overlapping ERG waves represent the unaltered response of the retina from the baseline as no significant difference was observed in the amplitude and latencies of ERG response at the various time point

The retina was evaluated for the lisinopril induced toxicity at 24, 48, 72 and 168 h by electroretinogram. No changes were observed in the ‘a’ and ‘b’ wave amplitude in the retinal response over the period of the study compared to their baseline ERG. Latencies of ‘a’ and ‘b’ wave were also not altered during the course of study following intravitreal injection (Fig. 4).

### Structural assessment of retina

**Fig. 5:**
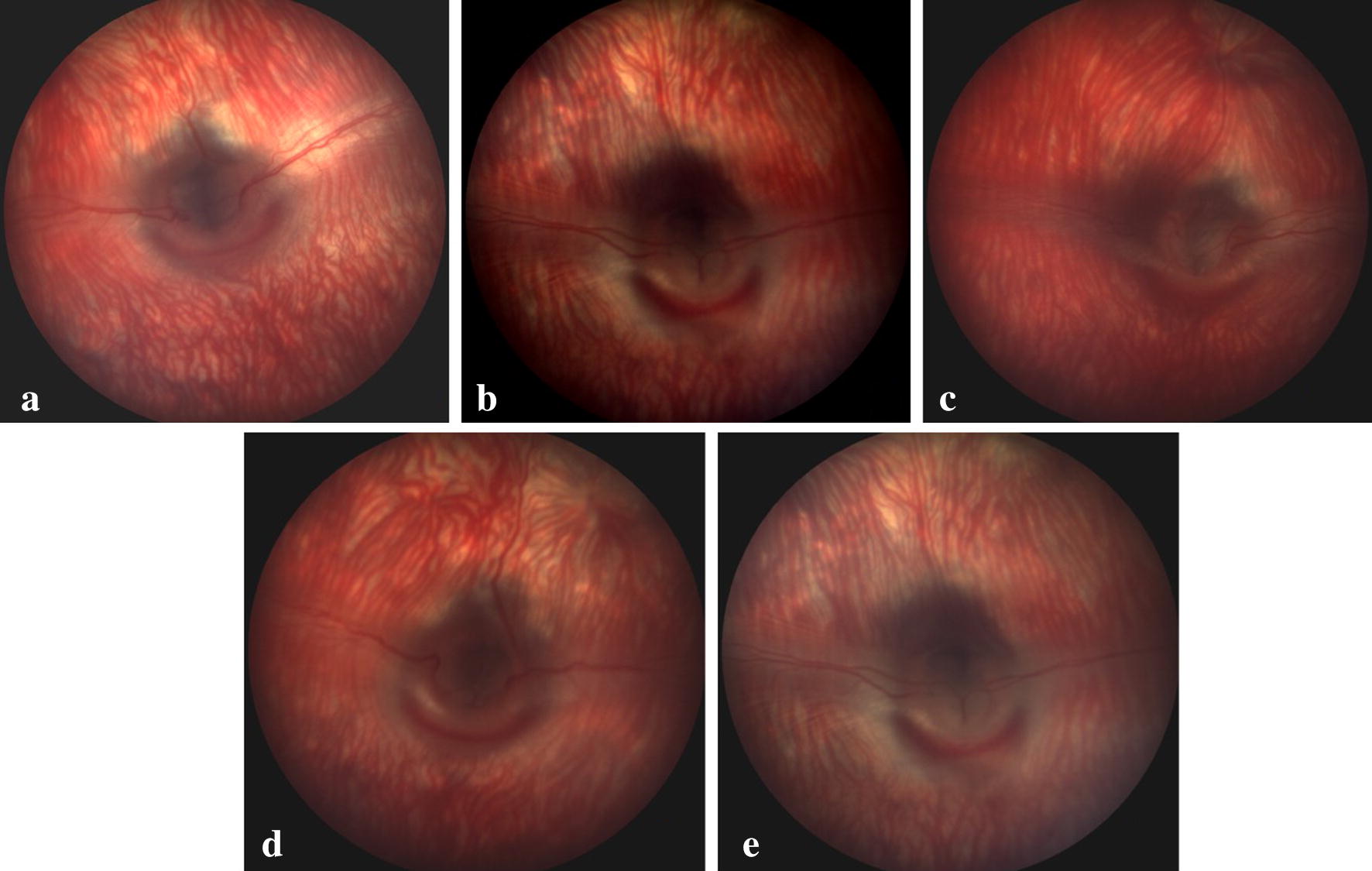
The study evaluated the retina for any possible toxicity for consecutive two vitreous half-life of lisinopril as calculated from the pharmacokinetic studies. The fundus photographs represent the fundus images of the injected eye at baseline, 24, 48, 72 and 168 h

The analysis of recorded fundus images of rabbits injected with intravitreal lisinopril at the dose of 40 µg/0.05 ml showed no significant morphological changes in the retina during the observational period of 7 days as evidenced by the analysis of the retinal structure and function (Fig. 5).

## Discussion

In 1987, Sramek et al., suggested that the ciliary body synthesizes renin and this renin may be part of an ocular Angiotensin II generating system [14]. Further, in a study done on the vitreous humour of diabetic retinopathy, it has been found that the pro-renin, relative to albumin and other plasma proteins, was higher in vitreous fluid from eyes with proliferative diabetic retinopathy complicated by traction retinal detachment than in eyes of nondiabetic subjects with spontaneous retinal detachment. From this study, the inference was made that pro-renin (and possibly renin) in the ocular fluid is controlled by an active and specific process, possibly local synthesis within the eye. In view of the vascular actions of angiotensin II, an intraocular renin-angiotensin system may play a role in diabetic retinopathy [15]. Lisinopril is the lysine analogue of enarprilate and an active constituent of the angiotensin converting enzyme inhibitors class. Lisinopril acts via inhibiting the angiotensin converting enzyme necessary for the conversion of angiotensin I into angiotensin II. Thus, indirectly inhibiting the vasoconstrictor activity of angiotensin II by regulating its production, as angiotensin II can stimulate the various factors including vascular endothelial growth factor and mediates angiogenesis [16].

In order to understand the role of retinal renin angiotensin system in the eye, Morvaski et al. (2000), have demonstrated that blockage of retinal renin angiotensin system might play as retinoprotective in the various vasoproliferative ocular pathologies [17]. Gilbert et al. (2000), also demonstrated that the possible role of the renin-angiotensin system in the vascular endothelial growth factor overexpression and hyperpermeability which accompany diabetic retinopathy and provide a potential mechanism for the beneficial effects of ACE inhibition in diabetic retinal disease [18].

Previous studies done in our lab has shown the presence multifold raised levels of renin angiotensin components in the vitreous of retinopathy of prematurity (ROP) patients and retinoprotective effect of the ACE inhibition in the animal model of ROP [8]. The study was an extension of the previous findings of our lab and thus the dose chosen was the effective dose found in our previous studies in the ROP model.

Lisinopril belongs to the category of angiotensin converting enzyme inhibitor and approved for oral use. For making an isotonic solution of lisinopril for intravitreal administration sterility is unavoidable. Therefore, this study has included thermal stability of lisinopril after autoclaving and from the results it is evident that it is unaffected by autoclaving thereby enabled accurate dosing to the rabbits.

The electroretinography studies following intravitreal injection of lisinopril have not shown any significant alteration in the ‘a’ and ‘b’ wave amplitude and latency, suggesting the safety of the injected molecule over the period of 7 days. On the fundus examination of the retina, no morphological changes were observed in the retina during the study. As electroretinography and morphological studies did not reveal any significant alteration from the baseline data, further studies to explore histological changes were abandoned.

The lisinopril concentration in the retina was detected above IC_50_ for blocking angiotensin converting enzyme for the period of 36 h. The maximum concentration at 1 h was not toxic to retinal tissues as evidenced by no observed changes in the ERG wave. The lisinopril localised delivery in the eye on the onset of ischemic retinopathies can be very beneficial in regulating the over-exaggerated retinal renin angiotensin system. Interestingly, the plasma concentration was found to be 167 fold less than the vitreous concentration. The ocular penetration study has shown, that the efflux of lisinopril in the systemic circulation reached to its maximum concentration 87 ng/ml (0.02 nM) at 1 h. As the observed concentration in systemic circulation was less than the IC50 of Lisinopril (1.2 nM), this mode of administration would be very helpful in avoiding the systemic side effects caused by this drug in the conditions like retinopathy of prematurity (ROP) in infants. The half-life after the single administration of the lisinopril in the vitreous humour was found to be 41.47 h, which was above the half-life of candesartan injected intravitreally as reported by Lee et al. [16]. The study evaluated the retina for any possible toxicity for consecutive two vitreous half-life of lisinopril as calculated from the pharmacokinetic studies. Therefore, a suitable drug delivery system as a vitreous insert for the controlled release for prolonged periods would be highly beneficial for conditions like ischemic retinopathy due to various conditions.

## Conclusion

To conclude, blocking retinal renin angiotensin delivery in the retina of lisinopril can be used as the targeted drug delivery approach for the infants affected from the ischemic retinopathy. The study observed that intravitreal injection of lisinopril did not any cause toxicity in rabbit retina as evidenced by no morphological or functional changes, moreover the efflux of drug in the systemic exposure of IVI lisinopril is very minimal. Further studies are required to evaluate the appropriateness of lisinopril as safe treatment modality for ROP infants.

## Abbreviations

ACE: Angiotensin converting enzyme
ACEI: Angiotensin converting enzyme inhibitor
RAS: Renin angiotensin system
LC-MS/MS: Liquid chromatography-tandem mass spectrometry
ERG: Electroretinogram
ROP: Retinopathy of prematurity

## References

[1] Yaguchi S, Ogawa Y, Shimmura S (2013). Angiotensin II type 1 receptor antagonist attenuates lacrimal gland, lung, and liver fibrosis in a murine model of chronic graft-versus-host disease. PLoS One.

[2] Sherwin JC, Kokavec J, Thornton SN (2015). Hydration, fluid regulation and the eye: in health and disease. Clin Exp Ophthalmol.

[3] Tenkumo K, Hirooka K, Sherajee SJ (2014). Effect of the renin inhibitor aliskiren against retinal ischemia-reperfusion injury. Exp Eye Res.

[4] Hatzopoulos KM, Vessey KA, Wilkinson-Berka JL, Fletcher EL (2014). The vasoneuronal effects of AT1 receptor blockade in a rat model of retinopathy of prematurity. Invest Ophthalmol Vis Sci.

[5] Kanda A, Noda K, Ishida S (2015). ATP6AP2/(pro)renin receptor contributes to glucose metabolism via stabilizing the pyruvate dehydrogenase E1 β subunit. J Biol Chem.

[6] Penno G, Chaturvedi N, Talmud PJ (1998). Effect of angiotensin-converting enzyme (ACE) gene polymorphism on progression of renal disease and the influence of ACE inhibition in IDDM patients: findings from the EUCLID randomized controlled trial. EURODIAB controlled trial of lisinopril in IDDM. Diabetes.

[7] Sjølie AK, Chaturvedi N (2002). The retinal renin-angiotensin system: implications for therapy in diabetic retinopathy. J Hum Hypertens..

[8] Nath M, Chandra P, Halder N (2016). Involvement of renin-angiotensin system in retinopathy of prematurity—a possible target for therapeutic intervention. PLoS One..

[9] Sarlos S, Wilkinson-Berka JL (2005). The renin-angiotensin system and the developing retinal vasculature. Invest Ophthalmol Vis Sci.

[10] Fletcher EL, Downie LE, Hatzopoulos K (2010). The significance of neuronal and glial cell changes in the rat retina during oxygen-induced retinopathy. Doc Ophthalmol.

[11] Van Kooij B, Fijnheer R, de Boer J (2006). A randomized, masked, cross-over trial of lisinopril for inflammatory macular edema. Am J Ophthalmol.

[12] Abel R, Boyle GL (1976). Dissecting ocular tissue for intraocular drug studies. Invest Ophthalmol.

[13] Gupta SK, Dhingra N, Velpandian T, Jaiswal J (2000). Efficacy of fluconazole and liposome entrapped fluconazole for C. albicans induced experimental mycotic endophthalmitis in rabbit eyes. Acta Ophthalmol Scand.

[14] Sramek SJ, Wallow IH, Day RP, Ehrlich EN (1988). Ocular renin-angiotensin: immunohistochemical evidence for the presence of prorenin in eye tissue. Invest Ophthalmol Vis Sci.

[15] Danser AH, Van den Dorpel MA, Deinum J (1989). Renin, prorenin, and immunoreactive renin in vitreous fluid from eyes with and without diabetic retinopathy. J Clin Endocrinol Metab.

[16] Lee JE, Lim DW, Park HJ (2011). Intraocular toxicity and pharmacokinetics of candesartan in a rabbit model. Invest Ophthalmol Vis Sci.

[17] Moravski CJ, Kelly DJ, Cooper ME (2000). Retinal neovascularization is prevented by blockade of the renin-angiotensin system. Hypertension.

[18] Gilbert RE, Kelly DJ, Cox AJ (2000). Angiotensin converting enzyme inhibition reduces retinal overexpression of vascular endothelial growth factor and hyperpermeability in experimental diabetes. Diabetologia.

